# Less is more: evaluating the impact of transitions of care pharmacist-led optimization on discharge antibiotic therapy duration in the emergency department

**DOI:** 10.1017/ash.2025.22

**Published:** 2025-02-26

**Authors:** Joel Zapata, Sandra Adeife, James B. Cutrell, Lindsay Jacobs, Marguerite L. Monogue, Michelle Ramos, James Sanders, Chen-Ching Wang, Esther Y. Golnabi

**Affiliations:** 1 Department of Pharmacy, University of Texas Southwestern Medical Center, Dallas, TX, USA; 2 Department of Internal Medicine, Division of Infectious Diseases and Geographic Medicine, University of Texas Southwestern Medical Center, Dallas, TX, USA

## Abstract

**Background:**

Patients discharged from emergency departments (ED) with antibiotics for common infections often receive unnecessarily prolonged durations, representing a target for transition of care (TOC) antimicrobial stewardship intervention.

**Methods:**

This study aimed to evaluate the effectiveness of TOC pharmacists’ review on decreasing the duration of discharge oral antibiotics in patients discharged from the ED at an academic medical center. Pharmacist interventions were guided by an antibiotic duration of therapy guidance focused on respiratory, urinary, and skin infections developed and implemented by the antimicrobial stewardship program. Pharmacist interventions from January 27, 2023, to December 29, 2023, were analyzed to quantify the total number of antibiotic days saved and the percentage of provider acceptance.

**Results:**

The ED TOC pharmacists reviewed a total of 157 oral antibiotic prescriptions. 86.6% percent of the reviews required pharmacist interventions. The most common indications for the discharge antibiotics were urinary tract infections (50.0%) and skin infections (23.4%). The total number of antibiotic days saved was 155 days with the provider acceptance rate of 76.5%. In 21% of cases, providers did not count the antibiotic doses administered in the ED, contributing to unnecessarily prolonged duration. 10.2% of patients re-presented to the ED while 6.4% of patients were hospitalized within 30 days of index ED discharge.

**Conclusion:**

The transitions of care pharmacist-led intervention was successful in optimizing the duration of discharge oral antibiotics in the ED utilizing prospective audit and feedback based on institutional guidance. The ED represents a high-yield setting for TOC-directed antimicrobial stewardship.

## Introduction

In 2022, healthcare professionals prescribed a total of 236.4 million outpatient antibiotic prescriptions.^1^ A major focus of the Centers of Disease Control and Prevention (CDC) guidance in recent years is antimicrobial stewardship that ensures appropriate and efficacious use of antibiotics to combat the urgent threat of antimicrobial resistance.^2^ A national analysis of emergency department (ED) visits with antibiotic prescribing during 2016–2021 concluded that 27.6% of antibiotics prescribed were inappropriate, which may be reduced through antimicrobial stewardship initiatives.^3^ Common inappropriate prescribing practices for antimicrobials include prolonged durations, antimicrobial selection deviating from national guidelines, and antibiotic initiation despite the absence of signs or symptoms of infections.^4^

A key antimicrobial stewardship intervention is pharmacist-led discharge stewardship which results in the optimization of antimicrobial selection and duration to support improved patient safety and outcomes.^5^ Transitions of care (TOC) pharmacists play a crucial role in ensuring optimal antimicrobial agents are used at appropriate doses as well as confirming proper duration of therapy.^6^

Our institution developed a standard duration of treatment guidance document for the management of common infectious syndromes to guide TOC pharmacists’ interventions to support optimization of duration of antibiotic therapy at the time of ED discharge. In this study, we aimed to determine the impact of the TOC pharmacist interventions on the duration of antibiotic therapy for patients discharged from our ED.

## Methods

### Duration of therapy guidance

The antimicrobial stewardship program (ASP) at the study institution developed an antimicrobial duration of therapy guidance document combining evidence-based literature and clinical expertise to optimize discharge oral antibiotic prescriptions in the ED for the following infections – respiratory tract infections, urinary tract infections, and skin and/or soft tissue infections (Supplementary Material). The infection type and drug selection were further subcategorized to provide specific recommendations for optimal drug, dose, and duration for a given infectious syndrome.

Prior to the initiation of the project in the ED, an infectious diseases (ID)-trained antimicrobial stewardship pharmacist provided an one-hour education session to the ED-specific TOC pharmacists on the duration of therapy guidance. None of the ED-specific TOC pharmacists were ID-trained. In addition, the guidance document was reviewed by ED physician leadership and presented at a monthly multidisciplinary ED clinical operations meeting. The document was forwarded to the ED medical director to disseminate to ED faculty via email.

### Intervention

Between January 27, 2023 and December 29, 2023, discharge antibiotic prescriptions in the ED were reviewed in real time by ED-specific TOC pharmacists using the ED track board monitoring system in Epic electronic medical record (EMR) software. ED-specific TOC pharmacists provide ED coverage between the hours of 2 PM and 12 AM daily. One TOC pharmacist per shift is on duty. Discharge antimicrobial prescriptions were reviewed for clinical accuracy, including appropriate selection based on indication, medication dose, frequency, and duration of therapy. If a prescription was deemed to be inappropriate by the TOC pharmacist, the pharmacist contacted the provider with the recommendation via Epic Secure Chat, telephone, or in person. Providers then accepted the intervention or rejected based on their clinical discretion considering patient factors and other diagnostic findings. After the provider was contacted, the intervention attempt was documented in the EMR. The TOC pharmacist noted if there were recommended changes to antimicrobial agent, duration of therapy, antimicrobial dose optimization or other antimicrobial recommendation(s). For three months following the initation of the project, interventions documented by the TOC pharmacists in the EMR were retrospectively reviewed by ID-trained antimicrobial stewardship pharmacist for appropriatness, and feedback was communicated to the TOC pharmacists via email as necessary.

### Study design

This retrospective descriptive study assessed the impact of the antibiotic stewardship intervention on duration of discharge antibiotics in the ED. Baseline demographic data, diagnoses (ie, indications for antibiotics), administered antibiotics in the ED, prescribed discharge antibiotics, duration of antibiotics pre-/post-intervention, and other types of pharmacist interventions (eg, medication counseling, dosing optimization, etc.) were collected using REDCap (Research Electronic Data Capture).^7^ During evaluation of the interventions, the indication for antibiotics was based on the study team’s assessment of labs, imaging, microbiologic cultures, and provider documentations in the EMR. The primary outcome of the study was the total number of antibiotic days saved, defined as the difference between the total duration initially prescribed (in ED and post-discharge) and the final actual duration prescribed at ED discharge. Secondary outcomes included rate of intervention acceptance by prescribing provider, repeat ED visit within 30 days of index discharge, and hospital admission within 30 days of index discharge. Reasons for 30-day ED visits and hospitalization were categorized as either related or unrelated to the originally diagnosed infection during index ED visits and were based on the study team’s assessment of labs, imaging, microbiologic cultures, and documentations in the EMR. Antibiotic therapy extension was defined as a case in which TOC pharmacist recommended extension of duration due to the initially prescribed duration being shorter than guidance-recommended duration.

## Results

A total of 157 discharge antibiotic prescriptions (in 157 unique patients) were reviewed during the study period. The median age of the population was 55 years old with 63.1% of the population being female (Table 1). The most common indications for antibiotics prescribed were urinary tract infections (50.0%), followed by skin and/or soft tissue infections (23.4%) and respiratory tract infections (13.3%). In patients treated for urinary tract infections, there were 8 patients who received treatment for what the study team deemed to be asymptomatic bacteriuria. The most common antibiotics prescribed at discharge were oral beta lactams (47.7%), followed by doxycycline (16.7%) and nitrofurantoin (14.9%) (Table 1).

**Table 1.** Demographics and clinical characteristics

Characteristic	N (%) (n = 157)
Age, median (IQR), years	55 (37–69)
Sex	
Male	58 (36.9)
Female	99 (63.1)
Race and ethnicity	
White	65 (41.4)
Black	61 (38.9)
Other or unknown	31 (19.7)
Indication for antibiotic prescribed^*^	
Urinary tract infection	79 (50.0)
Cystitis	48 (60.8)
Pyelonephritis	23 (29.1)
Asymptomatic bacteriuria	8 (10.1)
Skin and/or soft tissue infection	37 (23.4)
Non-purulent	24 (64.9)
Purulent	13 (35.1)
Respiratory tract infection	21 (13.3)
Community acquired pneumonia	12 (57.1)
Chronic obstructive pulmonary disease exacerbation	3 (14.3)
Others	6 (28.6)
Others^¥^	21 (13.3)
Discharge oral antibiotic prescribed	
Oral beta-lactams	83 (47.7)
Amoxicillin	6 (7.2)
Amoxicillin-clavulanate	7 (8.4)
Cefpodoxime	23 (27.7)
Cefuroxime	19 (22.9)
Cephalexin	28 (33.7)
Doxycycline	29 (16.7)
Nitrofurantoin	26 (14.9)
Fluoroquinolones	12 (6.9)
Azithromycin	8 (4.6)
Other^¶^	16 (9.2)

Abbreviations: IQR, interquartile range.

* One patient received antibiotic for two indications, making the sum of indications 158.

¥ Other indications: Sexually transmitted infections (4), conjunctivitis (4), dental infection (4), epididymitis (3), intraabdominal infections (3), paronychia (1), open wound with hematoma (1), puncture wound prophylaxis (1).

¶ Other antimicrobial prescribed: Trimethoprim-sulfamethoxazole (8), clindamycin (3), metronidazole (2), erythromycin (1), linezolid (1), fluconazole (1).

The total antibiotic days saved were 155 days with the pharmacist interventions (Table 2), and providers accepted 77% of interventions recommended by a TOC pharmacist (Table 3). Excluding antibiotic therapy extensions recommended by pharmacist (14 cases), the total antibiotic days saved were 203 days (Table 2). Of the cases requiring intervention, 86.6% of cases were due to the original duration of therapy prescribed being different from the guidance-recommended duration (Table 3). In 21.0% of cases, providers prescribed longer duration of therapy by not counting the antibiotic dose(s) administered in the ED (Table 3). Interventions were necessary but did not occur in 8 cases (5.9%) due to patients discharging before the intervention could take place (Table 3). Provider preference was the most common reason for rejecting pharmacist recommendations (54.2%; Table 3). Other interventions that TOC pharmacist performed included medication counseling (89.3%), antibiotic frequency and/or dose optimization (8.6%), and antibiotic selection optimization (2.1%).

**Table 2.** Clinical outcomes

Clinical Outcomes	N (%) (n = 157)
Total antibiotic days saved, days	155
Excluding antibiotic therapy extensions recommended by pharmacist, days	203
Emergency department re-visit within 30 days of index ED discharge	16 (10.2)
Reason for 30-day emergency department re-visit	
Related to original infection^*^	7 (43.8)
Unrelated to original infection and/or antibiotic prescribed	9 (56.3)
Hospital admission within 30 days of index ED discharge	10 (6.4)
Reason for 30-day hospital admission	
Related to original infection^¥^	6 (60.0)
Unrelated to original infection and/or antibiotic prescribed	4 (40.0)

Abbreviations: ED, emergency department.

* Reason for ED re-visit: Worsening/recurrent symptoms related to original infection (7).

¥ Reasons for hospital admission: ED cultures growing organisms resistant to discharge antibiotics and no oral alternatives existed and/or case required inpatient evaluation (3); worsening/recurrent symptoms related to original infection (3).

**Table 3.** Characterization of pharmacist interventions

Interventions	N (%) (n = 157)
Duration Intervention needed	
Yes	136 (86.6)
No	21 (13.4)
Provider acceptance rate	
Accepted	104 (76.5)
Rejected	24 (17.6)
Other: Patient discharged before intervention occurred	8 (5.9)
Reason for provider rejection	
Provider preference	13 (54.2)
Reason not documented by TOC pharmacist	8 (33.3)
No response received from provider	3 (12.5)
Types of interventions other than changes to duration of therapy	
Medication counseling	125 (89.3)
Antibiotic frequency and/or dose optimization	12 (8.6)
Antibiotic selection optimization	3 (2.1)
Provider counted emergency department-administered antibiotic dose(s) toward total duration of therapy	
Yes	28 (17.8)
No	33 (21.0)
Not applicable (ie, No antibiotic(s) administered in emergency department)	96 (61.2)

Abbreviations: TOC, transitions of care.

10.2% of patients re-presented to the ED within 30 days of index ED discharge (not leading to hospitalization) while 6.4% of patients were hospitalized within 30 days of index ED discharge (Table 2). Of the patients who either had ED re-visits or hospitalization within 30 days, 43.8% (7/16) of the ED re-visits and 60.0% (6/10) of hospitalizations were related to the original infection (Table 2). Three of the six hospitalizations occurred after the microbiologic cultures collected in the ED visit had resulted in isolated organisms that were resistant to initial discharge antibiotics (Table 2). The patients were contacted by the ED staff to return to the hospital and were admitted as there were no available oral antibiotic options and/or further in-hospital evaluation was needed.

## Discussion

In this intervention study, the implementation of a TOC pharmacist-led intervention on duration of therapy was associated with an avoidance of 155 days of total antibiotic therapy (1.0 day of antibiotic therapy saved per antibiotic order reviewed). Interventions made by TOC pharmacists in the ED were generally well accepted by providers with an acceptance rate of near 80%. The intervention project based on an institutional duration of therapy guidance document also characterized several other notable TOC pharmacist interventions, such as medication counseling and antimicrobial dose/frequency optimization, which have shown to reduce the odds of medication-related readmissions.^8^

Antibiotic stewardship services implemented at discharge provide a key opportunity to improve antimicrobial use and outcomes.^6,9–11^ In our study, approximately 87% of the discharge antibiotic prescriptions reviewed required interventions from TOC pharmacists, which is higher or comparable to results from previously published and similarly designed studies.^6,9^ Although we did not investigate the long-term adverse effects of unnecessary days of antibiotics, prolonged antibiotic therapy is associated with increased risks of *C. difficile*infections, antibiotic-related adverse events, and development of antibiotic resistance.^12–14^

We identified several areas of improvement to enhance our antimicrobial stewardship practice in the ED. Notably, urinary tract infections emerged as the most common indication associated with inappropriate prescribing. Our assessment found that eight out of 79 UTI cases (10.1%) were categorized as asymptomatic bacteriuria. Of these, five patients were still prescribed antibiotics inappropriately, indicating an opportunity for improvement in our ED. Additionally, we noted that a prevalent issue contributing to unnecessarily prolonged antibiotic therapy was the failure of providers to account for the ED-administered antibiotic doses in the total duration of therapy. In 54% of cases where antibiotics were administered in the ED before discharge, these doses were not included in the overall duration calculation. Accurate calculation of therapy duration is essential as each day of unnecessary antibiotic use can increase the risk of antibiotic-related adverse events.^12^

Several publications highlight the role of inpatient or TOC pharmacists in reviewing discharge antimicrobial orders.^5,6,9^ However, our study uniquely focused on ED-specific TOC pharmacist interventions. We chose to concentrate on particular infectious disease states, such as UTIs and skin and soft tissue infections, within the ED setting to assess the feasibility and impact of these pharmacist-led interventions. This targeted approach not only demonstrated the potential benefit of such interventions but also offers a model that could be applied even in resource-limited institutions.^15^ Moreover, this intervention study demonstrates the effective role that TOC pharmacists can play as antimicrobial stewardship extenders with education and guidance provided by antimicrobial stewardship pharmacists.

Limitations of this study include a relatively small sample size and the lack of comparison between pre-and post-implementation of the ED TOC pharmacist interventions. Additionally, potential intervention opportunities were missed during the hours when there was no ED TOC pharmacist coverage.

The TOC pharmacist-led intervention project, informed by an institutional-specific guidance document, successfully optimized the duration of discharge oral antibiotics in the ED. The substantial number of warranted interventions highlights that EDs are a ripe setting for pharmacist-directed antimicrobial stewardship. This project underscores the critical role TOC pharmacists can play in refining antibiotic use practices, especially at time of discharge, and suggests that EDs offer significant opportunities for antibiotic stewardship interventions.

## Supporting information

Zapata et al. supplementary material 1 Zapata et al. supplementary material

Zapata et al. supplementary material 2 Zapata et al. supplementary material

## Supplementary material

The supplementary material for this article can be found at https://doi.org/10.1017/ash.2025.22
