# Supplementary material for: Less is more: evaluating the impact of transitions of care pharmacist-led optimization on discharge antibiotic therapy duration in the emergency department
Source: Antimicrob Steward Healthc Epidemiol. 2025 Feb 26;5(1):e66. doi: 10.1017/ash.2025.22 (PMC11869057; doi:10.1017/ash.2025.22)
Supplement: Zapata et al. supplementary material 1 — Zapata et al. supplementary material [file S2732494X25000221sup001.docx]

**Duration of Antimicrobial Therapy at Discharge Guidance for Emergency Department**

Disclaimers:

1. The following guidance is based on available evidence (e.g., clinical practice guidelines and primary literature) and clinical judgment may supersede the recommendations of this guidance.
2. All medication regimens should be guided by cultures and susceptibilities, if available. Antimicrobial doses should be adjusted according to the recent, known renal/hepatic function.
3. If Infectious Diseases consultation was involved in this case, follow the ID provider recommendations over recommendations in this guidance.
4. Refer to the institutional Management of Beta-Lactam Allergy guidance to identify a potential alternative beta-lactam antibiotic for prescribing in cases where patients have documented drug allergies or intolerances to beta-lactam antibiotics.

|  | **Disease state** | **Antimicrobials** | **Duration** | **Reference** |
| --- | --- | --- | --- | --- |
| **Respiratory Tract Infections** | Community-acquired pneumonia | Preferred:   - Cefpodoxime 200 mg PO Q12H OR - Amoxicillin-clavulanate 875-125 mg PO Q12H   Alternative:   - PCN allergy: Levofloxacin 750 mg PO Q24H | **5 days** (if good clinical response and without immunocompromising condition or structural lung disease)  **7 days** (if good clinical response but with immunocompromising condition or structure lung disease) | Institutional CAP Guidance |
|  | Acute COPD exacerbation | Antibiotics should only be given to patients who have: increase in dyspnea, sputum purulence, and sputum volume  Preferred:   - Amoxicillin-clavulanate 875-125 mg PO Q12H   Alternatives:   - Azithromycin 500 mg on day 1, then 250 mg once daily on days 2-5 OR - Doxycycline 100 mg PO once daily | **5 days** | 2023 Global Strategy for Prevention, Diagnosis, and Management of COPD: 2023 Report |
|  | Influenza | Oseltamivir 75 mg PO twice daily   - Initiate within ≤ 2 days of symptom onset | **5 days**  May consider longer duration if immunocompromised | Clinical Practice Guidelines by the Infectious Diseases Society of America: 2018 Update on Diagnosis, Treatment, Chemoprophylaxis, and Institutional Outbreak Management of Seasonal Influenze |
| **Urinary Tract Infections** | Asymptomatic bacteriuria | Antibiotic treatment is recommended only in the following patients: 1) pregnant, 2) about to undergo urologic procedure with risk of mucosal bleeding, or 3) ≤ 60 days of renal transplant  Preferred:   - Cephalexin 500 mg PO four times daily OR - Nitrofurantoin 100 mg PO twice daily   Alternative:   - Fosfomycin 3 g packet PO once | Duration depends on antibiotic used; for complete guidance, refer to the Institutional UTI Guidance   - Cephalexin: 7 days - Nitrofurantoin: 5 days - Fosfomycin: 1 dose | Institutional UTI Guidance |
|  | Uncomplicated cystitis | Preferred:   - Nitrofurantoin 100 mg PO twice daily   Alternative:   - If CrCl <30 mL/min or intolerance/allergy to nitrofurantoin: Cefpodoxime 200 mg PO twice daily - If CrCl <30 and intolerance/allergy to nitrofurantoin AND severe beta-lactam allergy: Fosfomycin 3 g PO once | Duration depends on antibiotic used; for complete guidance, refer to the Institutional UTI Guidance   - Nitrofurantoin: 5 days - Cefpodoxime: 5 days - Fosfomycin: 1 dose | Institutional UTI Guidance |
|  | Uncomplicated pyelonephritis | Preferred:   - Levofloxacin 750 mg PO daily   Alternative:   - Cefpodoxime 400 mg PO twice daily | Duration depends on antibiotic used; for complete guidance, refer to the Institutional UTI Guidance   - Levofloxacin: 5-7 days - Cefpodoxime: 10-14 days | Institutional UTI Guidance |
|  | **Disease state** | **Antimicrobials** | **Duration** | **Reference** |
| **Skin and soft tissue infections** | Non-purulent (cellulitis) | Preferred:   - Cephalexin 1 g PO three times daily   If unable to receive cephalexin:   - Trimethoprim-sulfamethoxazole 2 DS PO twice daily | **5 days with prompt**  **clinical response** | Practice Guidelines for the Diagnosis and Management of Skin and Soft Tissue Infections: 2014 Update by the Infectious Diseases Society of America  McCreary EK, et al. Antibiotic myths for the infectious diseases clinician. *Clin Infect Dis*. 2023;77(8):1120-1125. |
|  | Purulent | - Trimethoprim-sulfamethoxazole 2 DS PO twice daily OR - Doxycycline 100 mg PO twice daily | **5 days with prompt**  **clinical response** |  |

**Definitions:**

- Asymptomatic bacteriuria
  - An isolation of bacteria in an appropriately collected urine sample from an individual not experiencing symptoms of urinary tract infection

**References (outside institutional syndromic guidances):**

Respiratory infections

1. Global strategy for the diagnosis, management, and prevention of Chronic Obstructive Pulmonary Disease (2023 Report). Global initiative for chronic obstructive lung disease. <https://goldcopd.org/2023-gold-report-2/>.
2. Uyeki TM, Bernstein HH, Bradley JS, et al. Clinical practice guidelines by the infectious diseases society of america: 2018 update on diagnosis, treatment, chemoprophylaxis, and institutional outbreak management of seasonal influenza. Clinical Infectious Diseases. 2019;68(6):e1-e47.

Urinary tract infections

1. Nicolle LE, Gupta K, Bradley SF, et al. Clinical Practice Guideline for the Management of Asymptomatic Bacteriuria: 2019 Update by the Infectious Diseases Society of America. Clin Infect Dis 2019; 68:e83.

Skin and soft tissue infections

1. Stevens DL, Bisno AL, Chambers HF, et al. Practice guidelines for the diagnosis and management of skin and soft tissue infections: 2014 update by the infectious diseases society of america. Clinical Infectious Diseases. 2014;59(2):e10-e52.
2. McCreary EK, Johnson MD, Jones TM, et al. Antibiotic myths for the infectious diseases clinician. Clinical Infectious Diseases. Published online June 13, 2023:ciad357.
